# Anti-Apoptotic Protein Bcl-xL Expression in the Midbrain Raphe Region Is Sensitive to Stress and Glucocorticoids

**DOI:** 10.1371/journal.pone.0143978

**Published:** 2015-12-01

**Authors:** Galina T. Shishkina, Tatyana S. Kalinina, Veta V. Bulygina, Dmitry A. Lanshakov, Ekaterina V. Babluk, Nikolay N. Dygalo

**Affiliations:** 1 The Federal Research Center Institute of Cytology and Genetics of Siberian Branch of the Russian Academy of Sciences, Novosibirsk, Russia; 2 Department of Physiology, Novosibirsk State University, Novosibirsk, Russia; Max-Delbrück Center for Molecular Medicine (MDC), GERMANY

## Abstract

Anti-apoptotic proteins are suggested to be important for the normal health of neurons and synapses as well as for resilience to stress. In order to determine whether stressful events may influence the expression of anti-apoptotic protein Bcl-xL in the midbrain and specifically in the midbrain serotonergic (5-HT) neurons involved in neurobehavioral responses to adverse stimuli, adult male rats were subjected to short-term or chronic forced swim stress. A short-term stress rapidly increased the midbrain *bcl-xl* mRNA levels and significantly elevated Bcl-xL immunoreactivity in the midbrain 5-HT cells. Stress-induced increase in glucocorticoid secretion was implicated in the observed effect. The levels of *bcl-xl* mRNA were decreased after stress when glucocorticoid elevation was inhibited by metyrapone (MET, 150 mg/kg), and this decrease was attenuated by glucocorticoid replacement with dexamethasone (DEX; 0.2 mg/kg). Both short-term stress and acute DEX administration, in parallel with Bcl-xL, caused a significant increase in *tph2* mRNA levels and slightly enhanced tryptophan hydroxylase immunoreactivity in the midbrain. The increasing effect on the *bcl-xl* expression was specific to the short-term stress. Forced swim repeated daily for 2 weeks led to a decrease in *bcl-xl* mRNA in the midbrain without any effects on the Bcl-xL protein expression in the 5-HT neurons. In chronically stressed animals, an increase in *tph2* gene expression was not associated with any changes in tryptophan hydroxylase protein levels. Our findings are the first to demonstrate that both short-term stress and acute glucocorticoid exposures induce Bcl-xL protein expression in the midbrain 5-HT neurons concomitantly with the activation of the 5-HT synthesis pathway in these neurons.

## Introduction

The world around us is not constant, and the organism has to adapt to changing conditions [1]. Multiple signaling pathways are involved in responses to stress, however the mechanisms underlying successful or not, adaptation to environmental perturbation, remain poorly understood. Despite the clear importance of the central serotoninergic (5-HT) neurotransmission in hormonal and behavioral responses to stress as well as in stress-related psychopathology [2–4], little is known about the adaptation of such indicator of 5-HT system activity as the rate-limiting enzyme in 5-HT synthesis tryptophan hydroxylase (TPH) [5] represented in the brain by the second variant, TPH2 [6, 7] to adverse events.

Numerous data indicate that gene and protein expression of TPH, the rate limiting enzyme for 5-HT synthesis, may be affected in the raphe nuclei, where cell bodies of 5-HT neurons are located, by stressful stimuli as well as by glucocorticoids, the levels of which are elevated under stress. However, these data are rather inconsistent. While some authors found no changes [8–11], the others reported an increase [8–10, 12–16], or decrease [17–21] in the enzyme expression and activity in the brain after exposure to stressors or glucocorticoids. The differences in TPH responses to these stimuli, in addition to dependence on the raphe region investigated, duration of stress exposure, genetic background and other experimental peculiarities [22, 23], may also be related to variations in expression of cell survival proteins. The study by McEuen et al. [10] showed that mice displaying signs of cell death in the raphe after chronic stress failed to exhibit the increases in TPH2 and anti-apoptotic factors during the stress experience found in mice resilient to stress. However, there is no available information on the effects of stress and glucocorticoids on anti-apoptotic proteins specifically in the 5-HT cells. Most investigations of the modulation of brain anti-apoptotic protein expression by these factors have been done in the hippocampus and cortical areas [24–31].

In order to determine whether stressful events affect anti-apoptotic protein expression in the raphe region and in the 5-HT cells, we focused on the Bcl-xL, which, in contrast to the second major anti-apoptotic protein, Bcl-2, continue to be highly expressed in adult brain [32] and was suggested to be crucial for the normal health of neurons and synapses [33]. We exposed adult male rats to the forced swim stress for the short-time (1–2 days) or prolonged (14 days) periods, and *bcl-xl* mRNA levels in the midbrain and anti-apoptotic protein immunoreactivities in the midbrain 5-HT cell bodies were assessed in stressed and control (unstressed) animals. To determine whether glucocorticoids may be implicated in stress effects on Bcl-xL, dexamethasone (DEX), a synthetic glucocorticoid acting predominantly through glucocorticoid receptors [34, 35], was administered alone or in combination with the glucocorticoid synthesis inhibitor metyrapone (MET) [36] that is used to prevent stress-induced glucocorticoid surge [37]. Finally, simultaneously with Bcl-xL, we evaluated effects of stress and glucocorticoids on TPH expression.

## Materials and Methods

### Animals

All animal use procedures were supervised and specifically approved by the ethic committee of the Institute of Cytology and Genetics in accordance with the guidelines of the Ministry of Public Health of Russia (supplement to order N 267 of June 19, 2003) and the European Council Directive (86/609/EEC). All efforts were made to minimize animal suffering and to reduce the number of animals used.

Adult males (2.5 months of age; experiments 1, 2 and 4) and neonatal pups (experiment 3) of Wistar rats were used in the present study. Adult male rats initially weighing 190–210 g were housed singly in polycarbonate cages (27.7 × 44 × 15 cm = w × l × h) with free access to food and water. Rat pups were with their mothers. The day of their birth was defined as a postnatal day 0. On postnatal day1, litters were culled to 8 pups. Animals were kept at temperature of 22–23° under natural illumination.

### Experimental procedures

#### Experiment 1

Adult male rats (n = 80) were exposed or not to short-term or chronic forced swim stress. For short-term stress, animals were forced to swim for 2 days, 15 min at the first day and 5 min at the second day according to published protocols [38]. For chronic stress, animals were exposed to the forced swim once daily (15 min) for 14 consecutive days. A significant (p < 0.05) increase in the relative adrenal weight (mg/100 g body weight) of chronically forced swim animals (19.59 ± 0.85) in comparison with unstressed rats (15.85 ± 1.23) is assumed to be an important indicator of chronic stress. Swim sessions were carried out between 1000 and 1200 h by placing rats in the swim tank (46 cm×20 cm glass cylinders) that were filled with water (24 ± 1°C) to a depth of 30 cm. After swimming, the rats were dried with towels and returned to their home cages. Rats were killed either 24 h after the first swim or 40 min, 2 h or 24 h after the second swim, or 24 h after the last swim session for 14 days (12–14 animals per group).

#### Experiment 2

Three groups of adult males (n = 30) were used. Animals of the first group remained untreated, whereas in other two groups, rats were injected subcutaneously (s.c.) with DEX (Krka, Slovenia; 0.2 mg/kg/0.4ml) or with an equivalent volume of vehicle (VEH; distilled water), and assessed at 6 h after the treatment (9–11 per group).

#### Experiment 3

Two-day old rat pups (n = 81) were used in this experiment. Pups from every litter were assigned to intact (2 pups), saline- (3 pups) or DEX-treated (3 pups) groups. Pups were injected with DEX (0.2 μg/g body weight, s.c.), with an equivalent volume (25 μl) of saline or remained untreated. The levels of *bcl-xl* mRNA were measured in the brainstem of these pups at 0 (intact), 0.5, 2, 4, 6, 8 and 24 h after treatment with saline or DEX (30 saline-treated rats sacrificed at all time-points were combined in a single saline group, and 5–7 DEX-treated animals were in each time-group).

#### Experiment 4

In this experiment, 25 adult rats were used (n = 4–6 per group). One group of animals remained without any treatment. Other rats were initially divided into two groups that received intraperitoneal (i.p.) injection of MET (2-methyl-1,2-di-3-pyridyl-1-propanone, Aldrich, USA; 150 mg/kg/4 ml) or an equivalent volume of vehicle (VEH; distilled water). Two hours after the first treatment, one half of MET-treated animals received subcutaneous (s.c.) injection of DEX (0.2 mg/kg/0.4ml) as well as one half of VEH-treated rats received the second injections of VEH by similar with DEX treatment route (s.c.). One hour after that, the rats from all treated groups: VEH i.p. (VEH1), MET i.p. (MET), VEH i.p.+VEH s.c. (VEH2), and MET i.p.+DEX s.c. (MET+DEX), and untreated animals were exposed to the first forced swim stress. Subcutaneous injections of DEX and VEH to animals from appropriate groups were repeated on the next day 1 h before the second forced swim stress exposures. As was shown previously, the dose of MET used had no appreciable effect on the basal corticosterone levels as well as all groups of rats that received DEX had lower corticosterone levels than the animals from other groups [39]. Animals were assessed for their *bcl-xl* mRNA levels at 2 h after the second swim.

### Analysis of messenger RNAs for *bcl-xl* and *tph2*

Following decapitation, the brains were quickly removed and the regions of the midbrain in adult animals using the atlas of Paxinos and Watson [40] and the brainstem in rat pups were rapidly isolated on ice and immediately frozen in liquid nitrogen. The midbrain sample from adult rats included the block of tissue from the rostral border of the superior colliculus to the rostral border of the pons and contained the DRN to approximately -8.7 mm bregma [15]. The brainstem sample from neonatal rats included the midbrain and pons.

The TaqMan® assay-based real-time PCR for genes was performed using TaqMan® Gene Expression Assays (*tph2*: Rn00598017_m1; *bcl-xl*: Rn00437783_m1; *beta-actin*: Rn00667869_m1; Applied Biosystems, Foster City, CA) and the ABI Prism 7000 Sequence Detection system (Applied Biosystems). All reactions were carried out in duplicate on cDNA samples in 96-well optical plates according to the manufacturer’s protocol in 25 μl of 1× TaqMan® Universal PCR Master Mix (Applied Biosystems). The real-time PCR consisted of one cycle of 50°C for 2 min and 95°C for 10 min, followed by 40 cycles each of 95°C for 15 s and 60°C for 1 min. The comparative ΔΔCT method was used to calculate mRNA expression relative to the beta-actin as an endogenous control according to manufacturer’s manual (Applied Biosystems).

### Double immunostaining for TPH and Bcl-xL

Four to six male rats were randomly selected from each group of the Experiments 1 and 2. Animals were deeply anesthetized, and their brains were removed after transcardial perfusion with ice-cold 0.9% physiological saline followed by 4% paraformaldehyde (PFA) in 0.1 M phosphate-buffer saline (PBS). The perfused brains were post-fixed in the same fixative at 4°C overnight and then cryoprotected by complete saturation in a 25% sucrose solution in 0.1 M phosphate buffer (PB; pH 7.4) at 4°C. After that, the brains were quickly frozen using powdered dry ice, cut into 16-μm-thick coronal sections on a cryostat and stored at −60°C before immunohistochemistry.

**Fig 1:**
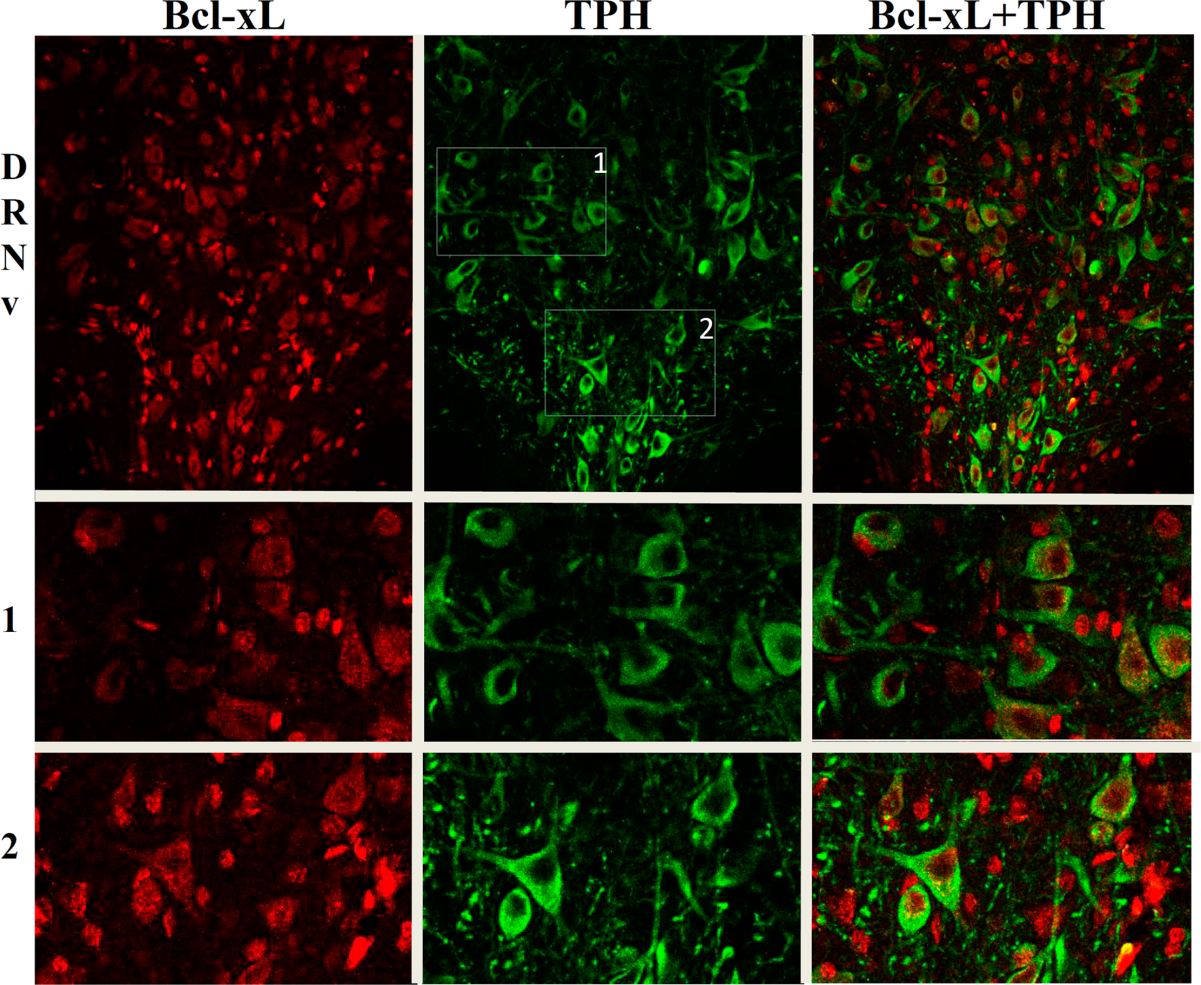
Representative photomicrographs of immunofluorescent staining. Bcl-xL (red), TPH (green) and double immunofluorescence for TPH and Bcl-xL are shown in the rat ventral part of the dorsal nucleus (DRNv), and at higher magnification in the random insets 1 and 2 as indicated in the DRNv TPH picture.

The sections were rinsed twice in 0.02 M PBS with 0.1% Triton X-100 (PBST), and nonspecific binding sites were blocked with a 5% normal goat serum in 0.02 M PBST for 1 h at room temperature. Subsequently, sections were incubated with primary antibodies against Bcl-xL (Santa Cruz sc-8392, mouse; dilution 1:200) and TPH (Millipore AB1541, sheep; dilution 1:200) for 24 h at 4°C. After that, the sections were washed with 0.02M PBS and incubated with secondary antibodies, goat anti-mouse IgG conjugated with DyLight 594 (115-515-207, Jackson ImmunoResearch; dilution 1:400) and donkey anti-sheep IgG conjugated with DyLight 488 (713-540-748, Jackson ImmunoResearch; dilution 1:400), for 4 h at room temperature. For the negative controls, sections were incubated without primary antibodies. All sections were then washed with 0.02 M PBS and mounted using Vectashield mounting medium with DAPI (Vector Laboratories, Burlingame, CA, USA). Images were acquired on a Zeiss (Germany) Axioskop 2 microscope equipped with the CCD camera, or on a confocal microscope (LSM 780 NLO) equipped with 405 nm, 488 nm and 561 nm lasers, using a Plan-Apochromat 20 objective (0.8 numerical aperture). TPH and Bcl-xL immunoreactivities were quantified by measuring their mean fluorescence intensities. To this end, fifteen to twenty well-defined TPH-positive cells were randomly chosen from each raphe region of interest (2–3 sections per rat), and the mean gray values of the selected cells from dorsal and ventral parts of the dorsal raphe nucleus (DRNd and DRNv, accordingly) and also from the median raphe nucleus (MRN) (from Bregma -7.64 to -8.30 mm; representative images are shown in Fig 1) were analyzed with the Zeiss Axiovision 4.8.2 or Zen software. The mean intensity of all images from each rat was averaged. The raphe regions for the protein expression analyses were determined using the atlas of Paxinos and Watson [39].

### Statistical analysis

The *bcl-xl* and *tph2* mRNA data were analyzed using a one-way ANOVA with *stress* or *drug* as a factor, followed by Bonfferoni’s post hoc test. Protein expression data were analyzed by two-way ANOVA with *raphe region* and *stress* as factors, followed by Bonfferoni’s post hoc test. The results were considered significant at probability level less than 0.05.

## Results

### Effects of the short-term and chronic forced swim stress on Bcl-xL and TPH expression in the raphe regions at 24 h after the last swim session

#### Bcl-xL

**Fig 2:**
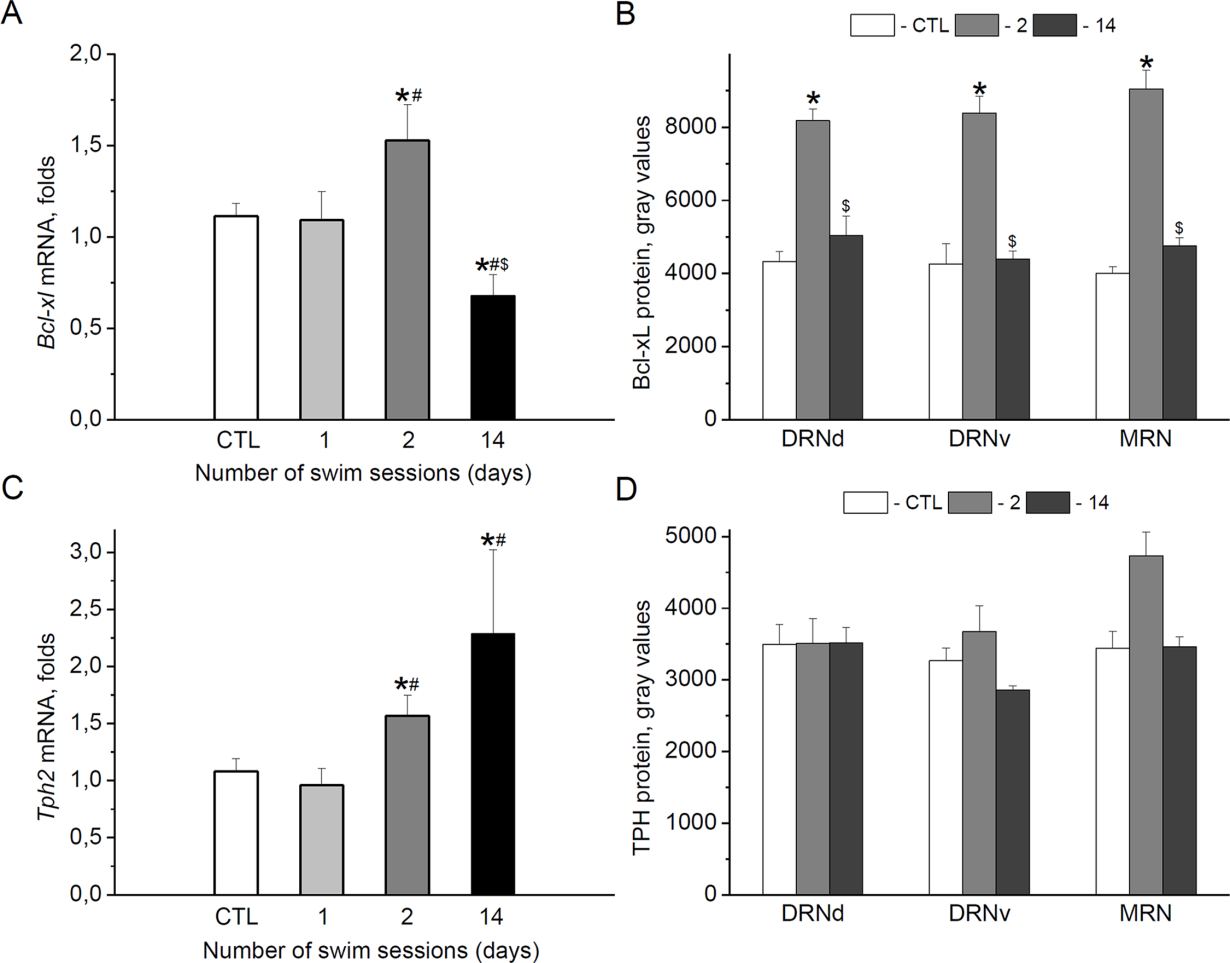
Effects of short-term and chronic swim stress on Bcl-xL and TPH. Bcl-xL (A, B) and TPH (C, D) mRNA (A, C) and protein (B, D) expressions in the midbrain (A, C) and 5-HT cell bodies (B,D) located in the dorsal (DRNd) and ventral (DRNv) parts of the dorsal raphe nucleus, and in the median raphe nucleus (MRN) were determined at 24 h after the last swim for 1 day (1), 2 days (2) or 14 days (14). *p < 0.05 vs. CTL (control unstressed aimals), ^#^p < 0.05 vs. 1, ^$^p < 0.05 vs. 2.

Compared with the control unstressed animals, when measured at 24 h after the last swim stress exposure, the midbrain bcl-xl mRNA level did not change after the first swim session, was significantly increased after the second swim and significantly decreased after chronic swim stress for 14 days [effect of *stress*: F(3,29) = 5.676, p < 0.01] (Fig 2A). Exposure of adult male rats to the forced swim stress also significantly affected Bcl-xL protein expression in 5-HT cell bodies located in both sub-regions of the dorsal raphe nucleus and in the MRN [effect of *stress*: F(2,39) = 93.754), p < 0.001] (Fig 2B). There was no effect of *raphe region* or interaction between factors. Bonfferoni’s post hoc test revealed an increase in anti-apoptotic immunoreactivities after the second swim in all regions. At the same time, no significant differences from controls were found for anti-apoptotic protein measured at 24 h after chronic stress.

#### TPH

Like *bcl-xl* mRNA, the midbrain *tph2* transcript levels showed significant increases at 24 h after the second swim. The increase in the enzyme gene expression was also observed in animals stressed for 14 days [effect of *stress*: F(3,28) = 5.015, p < 0.01] (Fig 2C). Two-way ANOVA revealed a significant effect of *stress* also on TPH protein expression in 5-HT cells [F(2,39) = 4.071, p < 0.05]. However, Bonfferoni’s post hoc test indicated a slight increase in TPH immunoreactivity at the level of tendency (p = 0.084) only in the MRN at 24 h after the second swim (Fig 2D).

### Bcl-xL and TPH expression at 40 min and 2 h after the second swim stress *mRNAs*

**Fig 3:**
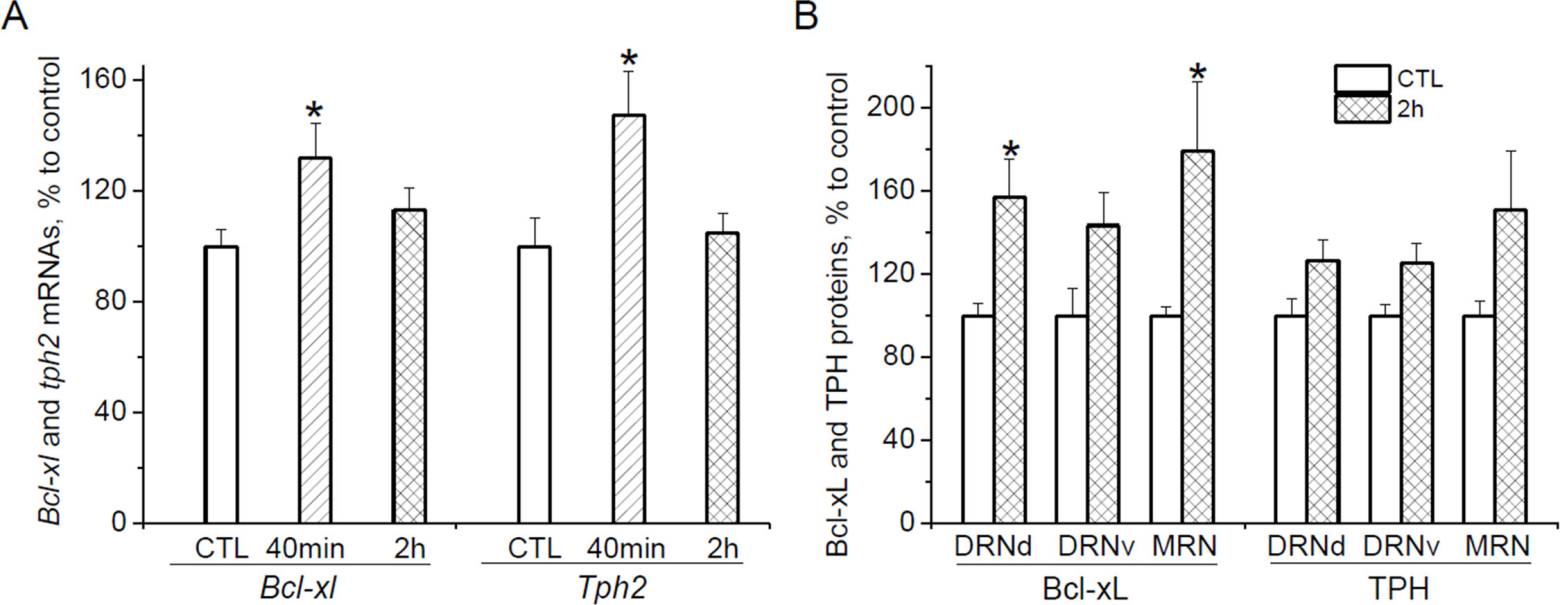
Bcl-xL and TPH expression at 40 min and 2 h after short-term swim stress. (A) *Bcl-xl* and *tph2* mRNA levels in the midbrain at 40 min and 2 h after the second forced swim. (B) Bcl-xL and TPH protein immunoreactivities in 5-HT cell bodies located in the dorsal (DRNd) and ventral (DRNv) parts of the dorsal raphe nucleus, and in the median raphe nucleus (MRN) at 2 h after the second forced swim. *p < 0.05 vs. CTL (control unstressed animals), ^#^p < 0.05 vs. 40 min.

To determine how soon after the second swim an up-regulation of gene expression occurred, *bcl-xl* and *tph2* mRNA levels were measured in the midbrain at the additional time points, 40 min and 2 h, after the second swim. Stress increased mRNA levels of both genes [effect of *stress* for *bcl-xl*: F(2, 26) = 3.451, p < 0.05, and for *tph2*: F(2, 26) = 4.890, p < 0.05] already at 40 min after the second swim (Fig 3A). Compared with unstressed animals, the increases at this time point were 31.9% (p < 0.05) and 47.3% (p < 0.05) for *bcl-xl* and *tph2*, accordingly. At 2 h after the second swim, *bcl-xl* and *tph2* mRNA levels were similar in rats that were forced to swim and unstressed control animals.

#### Proteins

The elevation in *bcl-xl* and *tph2* genes expression was accompanied by an increase of Bcl-xL and Tph2 immunoreactivity in the raphe regions at 2 h after the second forced swim (Fig 3B). Two-way ANOVA revealed a significant effects of *stress* on both proteins [F(1,29) = 23.435, p < 0.001 for Bcl-xL, and F(1,29) = 10.999, p < 0.01 for TPH], however according the Bonfferoni’s post hoc test, the differences between control and stressed animals did not reach statistical significance for TPH data.

### Effects of DEX on Bcl-xL and TPH protein expression

The fast responses of Bcl-xL and TPH to swim stress may be at least in part related to the action of glucocorticoids, the levels of which are significantly increased after the forced swim stress exposure [25, 41, 42]. To evaluate effects of glucocorticoids on Bcl-xL and TPH expressions, their mRNA levels and protein immunoreactivities were determined at 6 h after DEX treatment.

#### Bcl-xL

**Fig 4:**
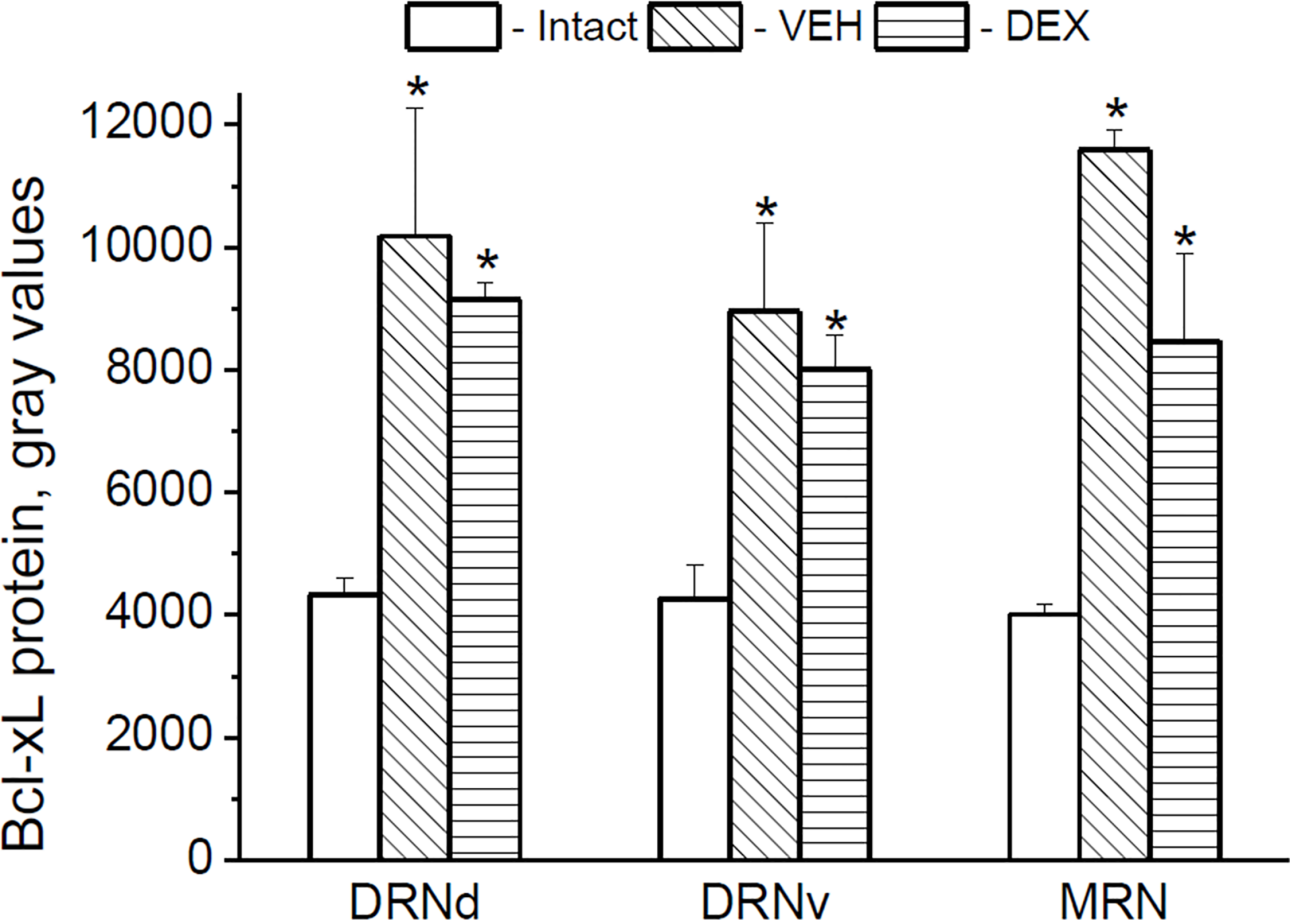
Effects of DEX on Bcl-xL in adult rats. Bcl-xL protein expression in the 5-HT cell bodies located in the dorsal (DRNd) and ventral (DRNv) parts of the dorsal raphe nucleus, and in the median raphe nucleus (MRN) at 6 h after the drug (VEH or DEX) injection. *p < 0.05 vs. Intact (untreated animals).

At 6 h after DEX treatment, the *bcl-xl* mRNA levels were similar in untreated (intact), VEH- and DEX-treated adult rats [effect of *drug*: F(2,20) = 0.980, p = 0.393] (data not show). At the same time, both VEH- and DEX-treated rats demonstrated increased Bcl-xL immunoreactivities in 5-HT cell bodies when compared with untreated animals in all investigated *raphe* regions (Fig 4) [effect of *drug*: [F(2,31) = 54.299, p < 0.001]. It is likely that endogenous glucocorticoid corticosterone, the level of which was shown to be markedly increased in adult rats already at 5 min after injection itself [43], may be implicated in VEH effects on Bcl-xL and masked the specific effect of injected DEX on this protein expression.

**Fig 5:**
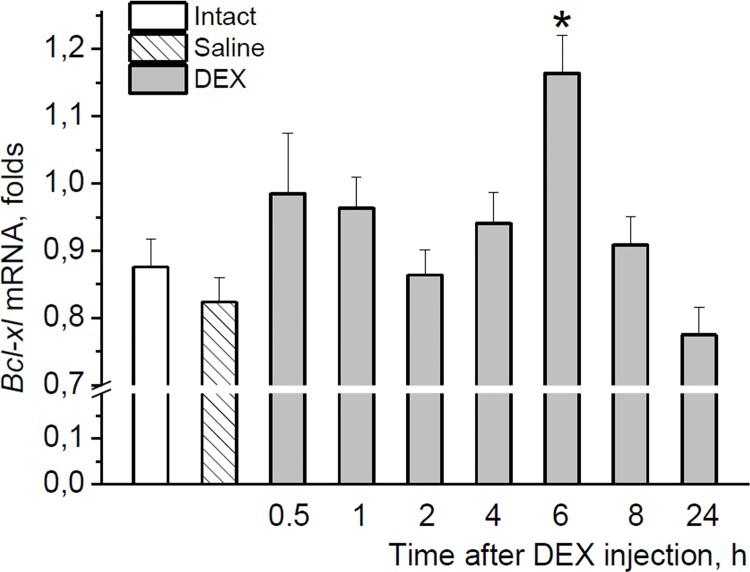
Effect of DEX on Bcl-xL in neonatal rats. The time-course of the neonatal brainstem *bcl-xl* mRNA response to DEX.*p < 0.05 with both Intact and Saline groups, and also with the values at 1 h, 2 h, 4 h, 8 h and 24 h after DEX.

To avoid possible impact of endogenous glucocorticoids on the Bcl-xL expression, we used neonatal rats. During neonatal development, rats have low levels of endogenous corticosterone which is relatively unresponsive to stressors [44, 45]. In this experiment, a single injection of DEX to neonatal pups caused a significant increase in the brainstem *bcl-xl* mRNA levels at 6 h after the treatment compared with both saline-treated and untreated controls [F(8,72) = 3.450, p < 0.01] (Fig 5). This increase was completely normalized during the subsequent 2 h. These data confirmed the ability of glucocorticoids to specifically impact *bcl-xl* gene expression.

#### TPH

**Fig 6:**
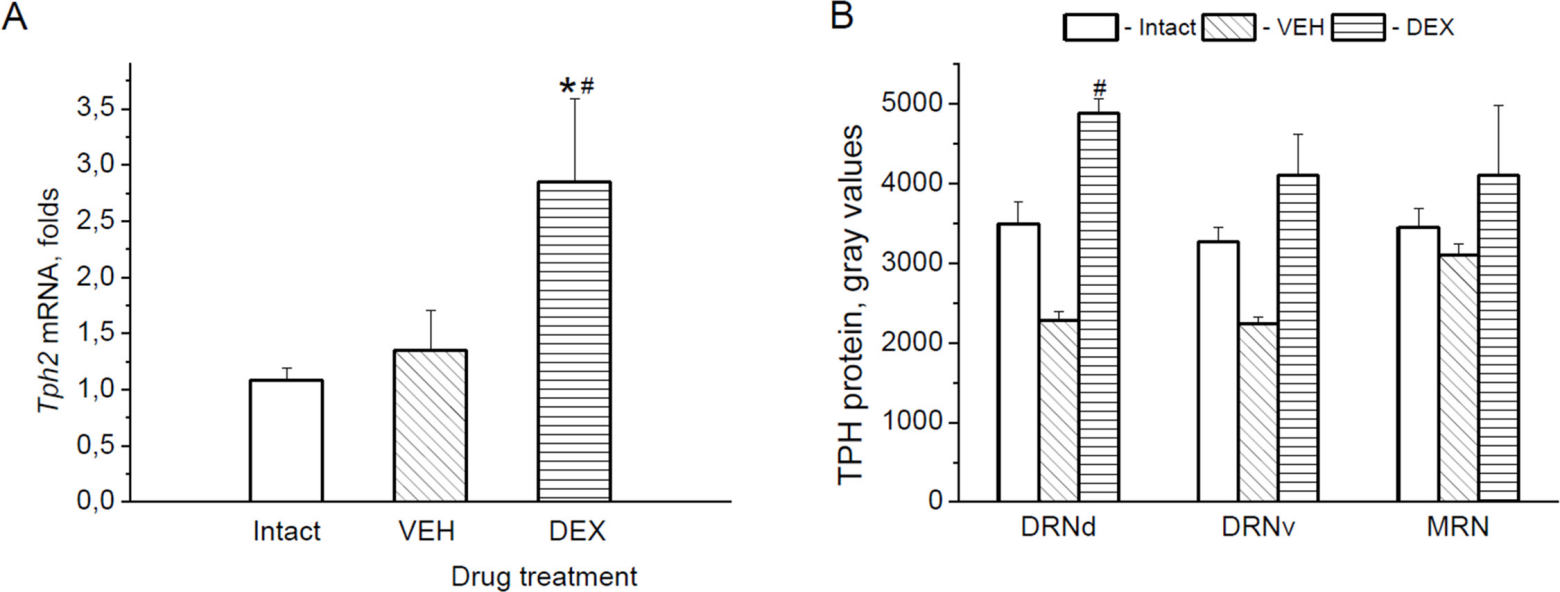
Effect of DEX on TPH. *Tph2* mRNA levels (A) in the midbrain and TPH protein (B) expression in the 5-HT cell bodies located in the dorsal (DRNd) and ventral (DRNv) parts of the dorsal raphe nucleus, and in the median raphe nucleus (MRN) at 6 h after the drug (VEH or DEX) injection. *p < 0.05 vs. Intact (untreated animals), ^#^p < 0.05 vs. VEH.

The level of the midbrain *tph2* mRNA was significantly increased by DEX as compared with saline and intact groups [F(2,22) = 6.176, p < 0.01] (Fig 6A). A two-way ANOVA of the enzyme protein levels also revealed the effect of drug treatment on TPH-immunoreactivities [F(2,31) = 7.955, p < 0.01] (Fig 6B). Bonfferoni’s post hoc test, however, showed statistically significant difference between saline- and DEX-treated animals only in the DRNd.

### The levels of the midbrain Bcl-xL mRNAs at 2 h after the second forced swim in rats injected with VEH, MET or MET+DEX

With the aim to determine whether glucocorticoids are indeed required for the acute swim stress-triggered increase in *bcl-xl* gene expression, we blocked the swim stress-induced elevation of glucocorticoid level by injection of MET 3 h before the first swim stress exposure with subsequent substitution of the hormonal signal by DEX administration 1 h before the first and second swim sessions.

**Fig 7:**
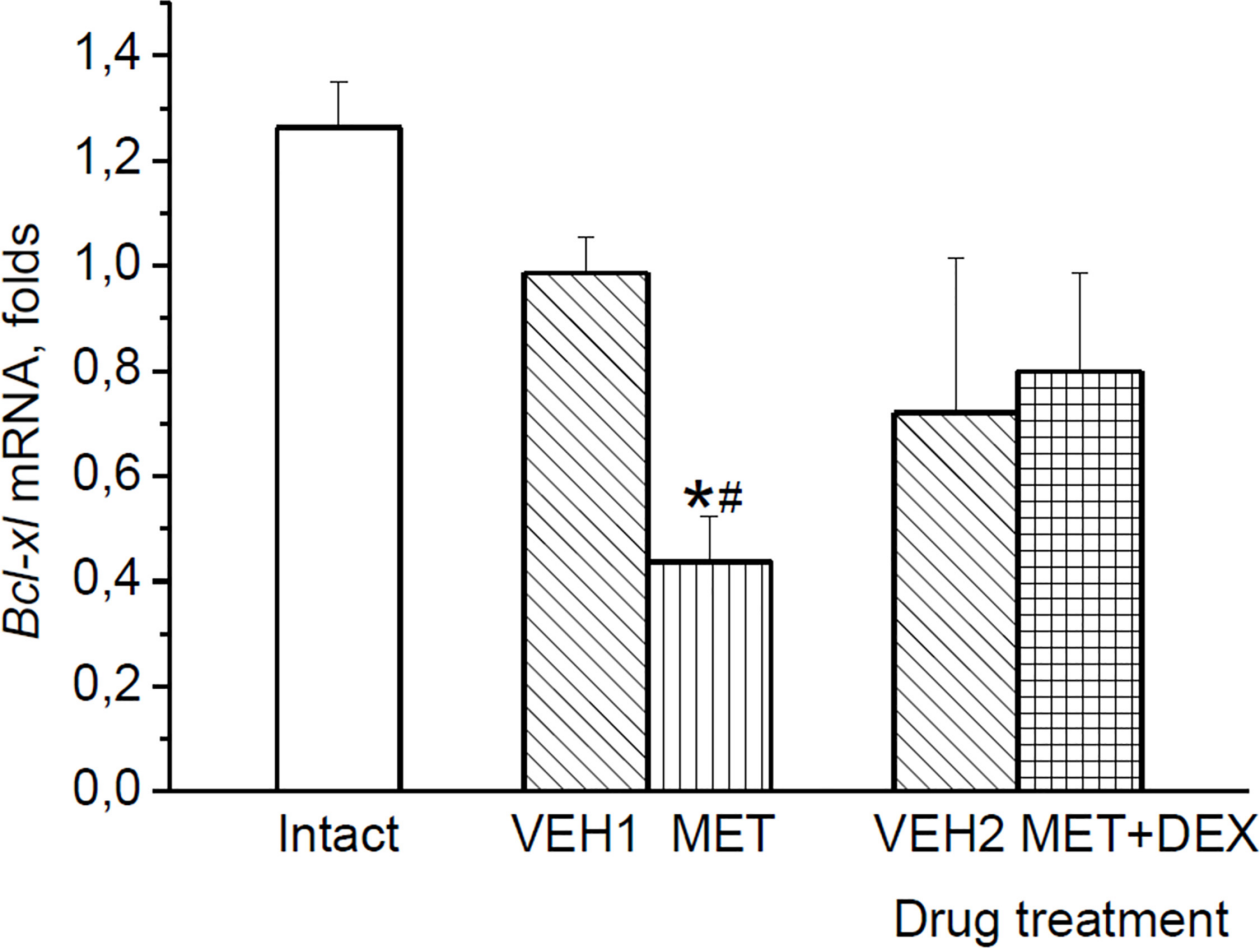
Effects of stress-induced glucocorticoid signal on Bcl-xL expression. The levels of the midbrain *bcl-xl* mRNA at 2 h after second forced swim: Stress-induced glucocorticoid signal was blocked by MET and substituted by DEX in MET+DEX treated animals. *p < 0.05 vs. Intact (untreated animals), ^#^p < 0.05 vs. VEH1.

Drug treatment had a significant impact on *bcl-xl* mRNA levels measured at 2 h after the second swim [effect of *drug*: F(4,20) = 6.454, p < 0.01] (Fig 7). When swim stress-induced increase in glucocorticoids was blocked by MET, the levels of *bcl-xl* mRNA were lower as compared with those in untreated and VEH-treated rats at 2 h after the second swim. Substitution of the MET-omitted glucocorticoid signal by DEX tended to restore the decreased by MET *bcl-xl* gene expression. The levels of *bcl-xl* mRNA in MET+DEX-treated animals were slightly higher than in MET-treated rats (p = 0.062) and did not differ from control animals.

## Discussion

Our findings are the first to demonstrate that stressful experiences can rapidly increase Bcl-xL protein expression in the cell bodies of 5-HT neurons. A significant up-regulation of anti-apoptotic protein was observed at 6 h even after a single vehicle injection (injection stress). Association of the increase in Bcl-xL protein expression with the induction of *bcl-xl* mRNA after the second swim links protein response to short-term stress with the activation of transcription. It should be noted that stress-induced Bcl-xL activation in cells labeled for TPH seems not to reflect false-positive identification of tyrosine hydroxylase (TH)-containing dopaminergic (DA) neurons, which have been reported in the rostral portion of the DRN [46]. Using the primary antibody solution, which contained antibodies, the same as in our study sheep anti-TPH, and mouse anti-TH, it was demonstrated that all DA neurons (immune-positive for TH) were immune-negative for TPH [47]. In one study, however, the sheep anti-TPH antibody (Chemicon International, Temecula, CA, USA) show a partial cross-reactivity to TH in a subset of TH-immunolabeled neurons that “were most prominent rostrally and dorsally in the DR” [48]. Interestingly that in this study [48], in rats that received a vehicle injection, a single swim stress produced a significant increase in the number of cells dually labeled with Fos and TPH at caudal levels in the DRN, both dorsally and ventrally, at 2 h after swim exposure, but did not change the number of cells dually labeled for Fos and TH.

The fast induction of *bcl-xl* mRNA by swim stress could result from glucocorticoid action. The levels of these hormones quickly increase after swim stress [25, 41, 42], and 5-HT neurons express substantial levels of glucocorticoid receptors [18, 49]. Glucocorticoid response elements were found within the *bcl-X* gene promoter regions [50, 51], and an increase in Bcl-xL expression at the protein and transcriptional levels mediated through glucocorticoid receptor signaling [52] was observed in C6 glioma cells after DEX treatment [52, 53]. In our study, the ability of DEX to up-regulate *bcl-xl* mRNA was demonstrated in the neonatal brainstem. In view of these data, the absence of reliable differences in *bcl-xl* mRNA levels between DEX and control groups of adult rats at 6 h after the treatment may be explained by that glucocorticoid-induced increase in bcl-xl mRNA levels seems to level off as it was found at 2 h after swim stress. In adult animals, Bcl-xL protein expression in 5-HT neurons was significantly increased after both DEX and vehicle injections. Activating effect of vehicle on Bcl-xL immunoreactivities similar to that of DEX is most likely related to the action of endogenous corticosterone, the level of which was shown to be markedly increased in adult rats by the injection itself already at 5 min [43]. This notion is supported by the absence of the effect of saline injection on *bcl-xl* mRNA in neonates, the hypothalamic-pituitary-adrenal axis of which is unable to increase corticosterone levels in response to stress [44, 45]. An implication of glucocorticoids in stress-induced activation of the *bcl-xl* expression in the midbrain was confirmed in experiments with MET. Prevention of the swim-induced increase in glucocorticoid levels by MET [37, 40] significantly decreased *bcl-xl* mRNA levels, whereas substitution of the MET-omitted glucocorticoid signal by DEX attenuated effect of MET on *bcl-xl* expression.

*Bcl-xl* gene expression in the midbrain demonstrated significant variations in the responses across swim stress sessions. Thus, *bcl-xl* mRNA levels did not differ from those in unstressed animals at 24 h after the first swim, were significantly increased after the second swim and decreased after the chronic forced swim. In previous studies, repeated unpredictable stress for 10 days [24] or 4 weeks [29] also led to a decrease in expression of *bcl-xl* in the other brain region, the hippocampus. The possible dependence of anti-apoptotic stress effects on the duration of stressor exposure is supported by data for other anti-apoptotic protein, Bcl-2. Thus, a significant decrease in hippocampal Bcl-2 protein expression was detected after stress for 2 [28] or 4 [54] weeks, whereas after stressful experiences for lesser periods, for example for 10 days, no changes [24] or even significant increases [27, 30, 55] were observed in both *bcl-2* gene and protein expression in the hippocampus.

The Bcl-xL up-regulation seen in the midbrain 5-HT cells following short-term swim stress or acute glucocorticoid administration may reflect an adaptive attempt of the brain to reduce potential apoptotic effects of these treatments on neurons. Signs of cell death were observed, for example, in the adult rat hippocampus not only after chronic stress, but also after 1 day of acute stress exposure [56]. Stressful events and glucocorticoids even at low doses often caused an increase in apoptosis as well as a decrease in the expression of Bcl-2 in the hippocampus of neonatal [57, 58] and adult [24, 28, 29] rats. However, numerous studies demonstrated that glucocorticoids also caused an increase in this anti-apoptotic protein expression in the hippocampus [27, 59], prefrontal cortex [26], and protected cells against death [31].

Activation of the anti-apoptotic protein expression by stress and glucocorticoids may be significant for preventing the development of stress-induced psychopathology. The ability to increase *bcl-xl* gene expression in the hippocampus in response to stress was associated with resistance to the development of stress-induced depression [25]. Stress- and/or glucocorticoid-induced expression of anti-apoptotic protein Bcl-xL may be particularly important for brain regions, where cell bodies of monoaminergic neurons involved in regulation of multiple physiological functions including behaviors, are located. It was found, for example, that rats kept for 6 weeks in constant darkness, in addition to behavioral features similar to depressed patients, have sensitized responses to subsequent stressors and increased apoptosis in the 5-HT raphe nuclei [60]. Animals with lower numbers of 5-HT neurons and *tph2* gene and protein expression in the DRN demonstrated more passive behavior in the forced swim test [61].

Numerous data, including the present results, show that the *tph2* gene expression can rapidly and in a flexible manner respond to stress and glucocorticoids and this may be important for adaptation to environmental challenges. For example, circadian peaks in raphe *tph2* gene expression and plasma corticosterone concentration occurred simultaneously. After the peak, the levels of the enzyme mRNA follow the decrease in the hormone concentrations and quickly dropped substantially within 4 h, at the first time-point assessed after the peak [7]. Circadian rhythm of *tph2* mRNA expression completely leveled by adrenalectomy and restored by corticosterone replacement [14]. Thus, stress-induced increase in glucocorticoid levels, evident at 40 min after the second swim [25], may contribute to the elevation in *tph2* gene expression at this time-point. At 2 h after swimming, when corticosterone levels returned to their basal values [25], *tph2* gene expression in rats exposed to swimming did not differ from that in unstressed animals in the present study. The subsequent increase in *tph2* mRNA levels observed at 24 h after the second, but not after the first, swim stress exposure was likely a secondary effect resulting from stress-induced prolonged changes in various neurobiological systems and transcription factors. Indeed, forced swim was shown to elicit biphasic (acute and long-lasting) increase in the phosphorylated cAMP response element-binding protein (pCREB) immunoreactivity in some brain regions [42]. An increase in expression of gene for CREB binding protein associated with the increase in TPH protein levels was observed in the rat dorsal raphe nucleus after chronic stress [9]. The suggested reasons for the observed time-course of the *tph2* mRNA levels after swim stress did not exclude any other explanations and precise mechanisms of this interesting phenomenon require further investigations.

The results of our study confirm sensitivity of TPH expression in the brain to stressful and glucocorticoid experiences that was demonstrated in most studies, although not in all [11]. Diverse effects of these treatments, an increase [8–10, 12–15, 62] or a decrease [17–21] in the enzyme protein levels or activities and/or mRNA levels were explained by different reasons including even species-dependence [22, 23] and as mentioned above, time of determination after the end of stress exposure. In the present study, the short-term swim stress exposure up-regulated TPH expression in the midbrain 5-HT neurons. The up-regulation occurred together with the activation of anti-apoptotic protein expression. Interestingly, in addition to the midbrain raphe nuclei, the main serotonergic region of the brain [7, 63], similar interrelation between the enzyme gene expression and cell-viability processes were found in the hippocampus [64]. In this study, using a whole transcriptome analysis, it was found that rats with low responsiveness to stress of a novel environment had increased hippocampal expression of *tph2* as well as of genes related to neuroprotection at 24 h following the second forced swim exposure.

The link between the decreased activity of the central 5-HT system and depression is supported by numerous data [65–67]. However, depressed state has been found to be accompanied by either an elevation in *tph2* gene [68] and TPH protein [69] expression or no changes in enzyme expression in the raphe nuclei [70]. In animal models, both pro-depressive and anti-depressant phenotypes were observed in mice with the deletion of *tph2* gene [71]. Important observations were also made using three genetically modified mouse models, all characterized by altered behaviors in depression-related paradigms, but demonstrated qualitative differences (significant decreases or increases) in *tph2* mRNA expression in the DRN [72]. It is therefore clear that the suggested relationship between TPH and depression may be rather indirect and may depend, for example, on different neurotransmitter systems [69], hippocampal neurogenesis [73] as well as on cell survival factors [74, 75].

Although further investigations are required, the results of the work hint at a possible link between expression of TPH2 and Bcl-xL in the midbrain during repeated stressful events. It may be possible that an increased TPH protein expression could reflect an acute protective action of the upregulated Bcl-xL on 5-HT cells after the second forced swim stress. During subsequent stressful events, the stress-induced augmentation of Bcl-xL expression disappeared due to yet unclear reasons. This may attenuate anti-apoptotic protective effect of Bcl-xL and lead to the weakening of serotonergic neuronal function evidenced by the activation of the *tph2* gene expression without an increase in TPH protein levels under chronic stress. Based on that stressful life events evoke neuronal cell loss associated with the development of psychopathology [76], the suggested relationship between Bcl-xL and TPH could contribute to the mechanisms of the resilience or vulnerability to stress and disorders related to degeneration of 5-HT neurons. Indeed, inability to induce expression of TPH2 and anti-apoptotic factors under stress was resulted in the raphe cell death and maladaptive behaviors [10]. In the view of these data, Bcl-xL-related pathways providing protection against pro-apoptotic action of stressful events and glucocorticoids may be important in elucidating the mechanisms of stress-related psychopathology.

In conclusion, the short-term stress and acute glucocorticoid exposure can induce Bcl-xL protein expression in the midbrain 5-HT cell bodies concomitantly with the activation of the 5-HT synthesis pathway in these neurons.
